# Beliefs and behaviours associated with vegetarian, vegan, and gluten-free diets among Canadians capable of bearing children

**DOI:** 10.1017/jns.2025.10044

**Published:** 2025-10-14

**Authors:** Olivia Morello, Erica Pellizzari, Madeline Erlich, Brenda Hartman

**Affiliations:** 1 School of Food and Nutritional Sciences, Brescia University College, London, ON, Canada; 2 Department of Nutritional Sciences, Faculty of Medicine, University of Toronto, Toronto, ON, Canada; 3 Toronto 3D Knowledge Synthesis and Clinical Trials Unit, Clinical Nutrition and Risk Factor Modification Centre, St. Michael’s Hospital, Toronto, ON, Canada; 4 Brescia School of Food and Nutritional Sciences, Western University, London, ON, Canada

**Keywords:** vegetarian, vegan, gluten-free, beliefs, behaviours, Canadians, CCBC, Canadians capable of bearing children, GF, gluten-free, CCHS, Canadian community health survey, CD, celiac disease, FFQ, food frequency questionnaire, GI, gastrointestinal, ADA, American dietetic association

## Abstract

There is increased interest in self-selected exclusionary diet patterns, specifically vegetarian, vegan, and gluten-free (GF) diets, but there is a lack of research exploring the beliefs and behaviours surrounding these diets in Canadians capable of bearing children (CCBC). The goal of this study was to explore the beliefs and behaviours of CCBC who follow vegetarian, vegan, and/or GF diets using mixed methods. A self-administered online Qualtrics™ survey containing 102 questions was conducted using open text and closed format questions. Continuous variables were summarized using mean and standard deviation while percentages were used to summarize categorical variables. Qualitative data was analysed using thematic analysis. A total of 271 CCBC between 18–45 years of age were analysed, with 27%, 22%, and 3.7% indicating they followed a vegan, vegetarian, and/or GF diet, respectively. Three main themes emerged that influenced CCBC beliefs about their chosen diet. The belief that these diets are healthy or could impart health in some way, was the main reason for following their chosen diet, especially in those who identified as vegetarian. Ethical/moral concerns, primarily around animal welfare and the environment, was the second theme for following their chosen dietary pattern, especially amongst those who identified as vegan. Perception of social judgement in the forms of criticism, guilt, and isolation were noted by some CCBC, with family, friends, and colleagues interacting differently with them because of their dietary choices. These findings serve to enhance our understanding of the beliefs and behaviours of CCBC who choose to follow exclusionary diets.

## Introduction

Interest in self-selected exclusionary diets, such as vegetarian, vegan, and gluten-free (GF) has increased in recent years,^(1)^ and Canadians are no exception to this, with 2.3 million identifying as vegetarian in 2022—a stark increase from 15 years ago, when only 900,000 identified as vegetarian.^(2)^ Adding an additional 850,000 Canadians who indicated that they follow a vegan diet, an estimated 6–9% of the Canadian population identifies as vegetarian or vegan.^(3)^ A vegetarian diet is defined as a diet that contains vegetables, fruits, grains, nuts, seeds, and legumes.^(4–6)^ In addition, the diet may include products not derived from animal slaughter such as eggs, milk, and cheese.^(4–6)^ While several definitions of vegan exist, a vegan diet is typically defined as not only excluding products from animal slaughter but also excludes eggs, dairy, and sometimes honey.^(4,5)^ Importantly, vegans identify veganism as a philosophical stance and way of living that rejects the use of animals as a commodity in any form, as opposed to just a diet, and make efforts to exclude anything that exploits animals including types of clothing (leather, wool, silk), cosmetics, cleaning products etc., and will even avoid institutions such as zoos.^(7,8)^ Currently, there is no standard definition of ‘plant-based’ dietary patterns, but these are considered to exclude or mostly exclude foods of animal origin.^(9)^

Fewer Canadians choose to follow a GF diet (which is defined as eliminating any gluten protein or modified gluten protein from barley, wheat, kamut, spelt, triticale, oats or rye),^(10)^ with a prevalence rate of only 1.9% based on data from the 2015 Canadian Community Health Survey (CCHS)—Nutrition.^(11)^ It is important to note that this estimate does not distinguish between Canadians following a GF diet for medical reasons (such as a diagnosis of celiac disease [CD]) or non-medical reasons.

While the CCHS-Nutrition provides a snapshot of the rates of individuals who avoid certain foods, it is not designed to provide more detailed information about factors related to the decisions and habits surrounding the chosen dietary pattern. Understanding these factors can help identify potential areas of support, like nutrition education, for those who do follow these exclusionary diet patterns, especially those individuals in their reproductive years.

Throughout this article, the term Canadians capable of bearing children (CCBC) is used to describe individuals who can become pregnant. The majority of people who have a uterus and can become pregnant are those who are ‘assigned female at birth’ and identify as female but people of all genders are capable of bearing children.^(12)^ Identifying factors that influence CCBC decision-making around their exclusionary diet patterns is particularly important considering that those who identify as female are more likely to follow a vegetarian, vegan,^(2,13)^ and/or GF diet^(11)^ compared to those who identify as male. Those following a vegetarian/vegan diet in Canada, are more likely (63%) to be in their prime reproductive years under the age of forty.^(2)^ Healthcare professionals whose patients are comprised of CCBC following these diets may be concerned about deficiency or sub-optimal nutrient status. This is because, if not properly managed, these diets may lack nutrients,^(14–17)^ like the one-carbon nutrients folate (found in folate-fortified grain products), choline, and vitamins B_6_ and B_12_ (found primarily in animal-based foods) that are key during reproduction to promote normal growth and development.^(18)^ Furthermore, these one-carbon nutrients been shown to be critical for foetal neurodevelopment and birth defect prevention.^(18)^ It is also important to note that consumers in general, including CCBC, are more likely to derive their nutrition information from online sources, including social media, where the quality and accuracy of information is often low.^(19)^

The decision to follow self-selected exclusionary diet patterns is often a personal choice. Common reasons given for those following a vegetarian and vegan diets include personal health, the environment and sustainability, animal welfare, ethics, and religion,^(7,20–22)^ while a healthier and more nutritious diet is often mentioned as the reason to avoid gluten.^(23,24)^ A recent study examining the body of vegetarian/vegan literature found that health (83%), environmental benefits (75%), and animal related concerns (67%) were considered the central motives found in the various papers within this area of study.^(22)^

Motives provide insight into our beliefs and can serve to inform choices made around exclusionary diets by an individual. Literature to date has predominantly focused on behaviours in the context of dietary adherence. Rothgerber^(25)^ found vegans are more likely to be motivated by ethical concerns compared to those who follow a plant-based dietary pattern who are motivated by health. A desire to maintain social cohesion may also motivate individuals identifying as vegetarian to consume meat on occasion.^(26)^ In the case of a GF diet, a diagnosis of CD increases dietary adherence as compared to those following the diet without CD.^(23)^ Furthermore, those diagnosed with CD pay more attention to food and ingredients, ate more meals at home compared to fast food or meals at restaurant.^(23)^

While the above findings are important, they do not necessarily explore the unique impacts that following an exclusionary diet can have on one’s life and habits and how those in their prime reproductive years, like CCBC, may be affected. There has been a recent increase in the literature examining the impact of vegetarian/vegan diets during pregnancy and whether the diet(s) are nutritionally adequate or they focus on the link between diet and maternal/foetal outcomes.^(14–17,27)^ However, there is little research looking at diet patterns, and the factors that determine those patterns, in the reproductive years outside of the state of pregnancy. To facilitate a deeper understanding of these impacts, it is necessary to survey and quantitatively and qualitatively assess the explicit beliefs held by CCBC from across Canada and their food-related behaviours. The aim of this study then, was to explore the beliefs and behaviours of CCBC who follow vegetarian, vegan, and/or GF diets.

## Materials and methods

### Data collection

To explore the beliefs and behaviours of CCBC who follow self-selected exclusionary diet patterns, an anonymous web-based survey was designed and distributed using the secure Qualtrics™ (Provo, UT) platform. Quatrics^TM^ is compliant with ISO27001, FEDRAMP, and other industry standards to maintain data security (Provo, UT). The cross-sectional survey contained 102 questions, and used a combination of closed format, open-ended, and open text questions that were split into seven sections: (A) socioeconomic and demographic (8 questions); (B) general medical history (12 questions); (C) diet patterns and beliefs (13 questions); (D) diet adherence and behaviours (13 questions); (E) dietary information and nutrition knowledge check (7 questions); (F) supplement use (13 questions); (G) food recall (34 questions). No anthropometric data was collected. The survey took about 30–35 minutes to complete. As an incentive to complete the survey, participants were invited to participate in a raffle for a $50 Amazon gift card. If they were interested in the raffle, they were then sent to a separate survey link. A third party administered the draw for the gift card to maintain participants’ anonymity. Sections E-G of the survey, which collected the information on one-carbon nutrients knowledge and beliefs, are discussed separately.^(28)^

A literature search through OMNI and PubMed databases from June to August 2020 was completed to aid in developing the questions. The survey was pilot tested by a small convenience sample to assess survey comprehension, flow, and time needed to complete the survey prior to administration. It was then posted in May 2022 in nine Facebook groups (Toronto Vegans, Vegan, and Gluten Free, PlentyofVegans (Vegan Singles of Toronto and area GTA CANADA), Vegans in Hamilton Ontario, Vegetarians and Vegans in Guelph, Ontario Canada, Toronto Vegetarians Association, Plant-based Vancouver, Vegans and Vegetarians of Alberta, Halifax Vegetarians/Vegans). To be eligible to participate in the study, individuals were required to be Canadian citizens between the ages of 18 and 45 who were capable of bearing children. This study was conducted according to the guidelines laid down in the Declaration of Helsinki, and all procedures involving human subjects were approved by Brescia’s Research Ethics Board and has since been transferred to Western University. Written informed consent was obtained from all participants before participants were allowed to complete the self-administered survey.

### Sample size and data clearance

There are between 7 and 8 million Canadian women between the ages of 18–45^(29)^ and it is estimated that 6–9% of the Canadian population follow a vegetarian and vegan diet.^(2)^ Thus, it was estimated that about 705,000 Canadian women would be vegetarian/vegan. The final sample size was calculated using the Qualtrics™ sample size calculator with a 90% confidence level and a 5% margin of error giving us a minimum sample size of 271 CCBC who follow a vegan, vegetarian, and/or GF diet.

**Figure 1.**
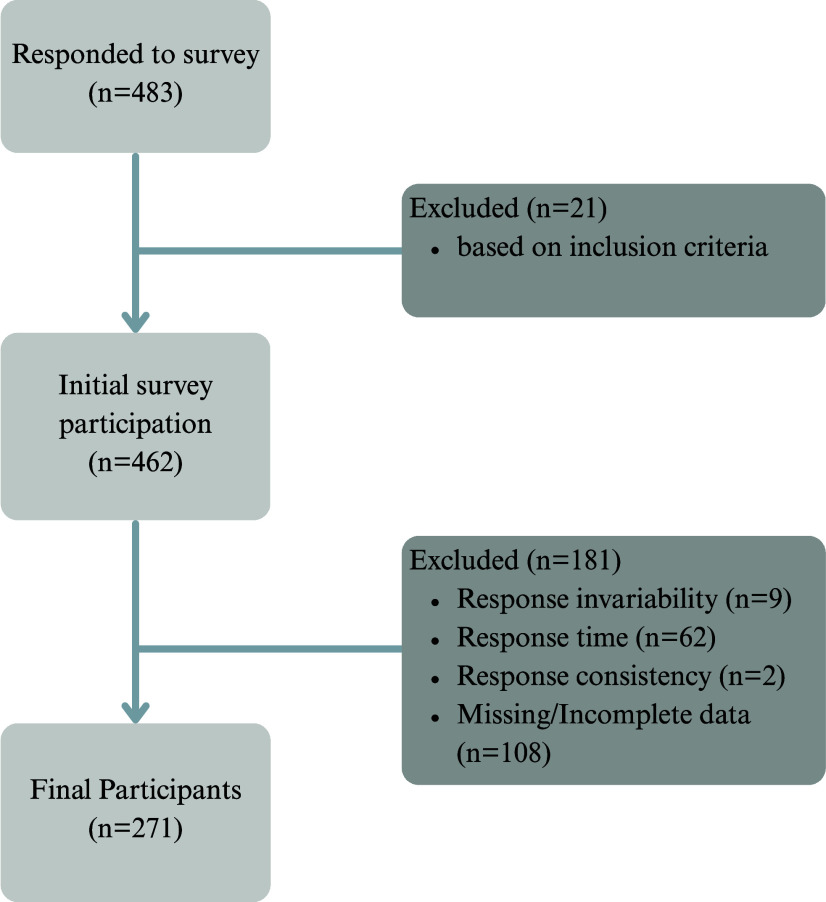
Breakdown of data clearance of survey responses to final number. *N* = 271.

Data clearance was completed by one author (E.P.). Several measures were used to assess the credibility of survey responses. The survey included questions designed to check for consistency in the answers to help ensure the integrity of the data collected. Other measures used to assess credibility included response invariability (selecting similar responses for dissimilar questions), response time (total time taken to complete the survey from start to finish), and response consistency (contradiction in responses throughout the survey) indirectly indicated careless responding and were grounds for removal of data.^(30)^ Direct measures of careless responding included missing or incomplete survey data, defined as <50% of the survey completed, as well as responses that did not meet the inclusion criteria (e.g. participant <18 years old or >45 years old). See Figure 1.

### Data analysis

The study used a mixed methods approach. Data were exported from Qualtrics™ into SPSS version 26.0 (IBM SPSS Statistics for Windows, Armonk, NY: IBM Corp). Continuous variables were summarized using the mean and standard deviation (SD) while percentages were used to summarize categorical variables. Qualitative data was analysed using thematic analysis.^(31)^ Two authors (O.M. and E.P.) completed qualitative analysis separately using the steps described by Braun and Clarke starting with familiarization.^(31)^ Initial codes were then generated, followed by thematic mapping of the codes into potential themes. Themes were reviewed in relation to the individual codes and to each other to ensure coherence within the dataset. Revisions to codes and/or themes were made as necessary. Themes were then identified and named and compared to ensure consensus between all authors.

## Results

### Participant characteristics

A total of 483 participants responded to the survey. Participants were removed if they did not meet the inclusion data or responded carelessly leaving 271 who completed the survey. See Figure 1. More vegans than vegetarians responded although 41% did not specify their diet at all (Table 1). The majority of the CCBC were under the age of 39 (89%) with a mean age of 29.5 ± 6.1 years and had incomes greater than $60,000 CAD per year. Almost one quarter of the vegan group were at least forty years of age. Almost 24% of the respondents indicated they were either pregnant or breastfeeding when the survey was conducted. More than half the respondents reported not smoking or using cannabis in any form. Alcohol use followed a similar pattern with almost 75% of the respondents having at most only one drink a week or never drinking at all. Survey respondent characteristics are included in Table 1.

**Table 1.** Socio-demographic characteristics of Canadians capable of bearing children survey respondents (*n* = 271)

Characteristics	Total		mean	SD	Veg		Vegan		GF	
	*n*	*%*			*n*	*%*	*n*	*%*	*n*	*%*
**Age (y)**										
18–29	140	51.7	25.3	2.5	34	57.6	39	45.9	5	50.0
30–39	101	37.3	32.9	2.9	20	33.9	25	29.4	5	50.0
40–45	30	11.1	42.3	1.8	5	8.5	21	24.7		
**Diet**										
Vegan	75	27.7								
Vegetarian	60	22.1								
Gluten Free	10	3.7								
Vegan and Gluten Free	7	2.2								
Vegetarian and Gluten Free	2	< 0.01								
Plant-based	5	1.8								
Not Provided	112	41.2								
**Education**										
High School	27	10.0			2	3.4	12	3.4	1	10.0
College/University	166	61.3			43	72.9	54	63.5	7	70.0
Graduate Degree	72	26.6			14	23.7	15	17.6	2	20.0
Professional/Doctoral Degree	6	2.2			0	0	4	4.7	0	0
**Have you ever completed any nutrition-related education?**										
No	111	41.0			24	40.7	52	61.2	2	20.0
Yes	157	58.0			35	59.3	33	38.8	8	80.0
Not Provided	3	1.0								
Ethnic Background*										
Canadian	142	42.0			28	47.5	45	52.9	9	90.0
British Isles	127	37.6			14	23.7	21	24.7	1	10.0
French	9	2.7			3	5.1	2	2.4		
Other European	7	1.8			2	3.4	3	3.5		
Asian	14	4.5			2	3.4	5	5.9		
South Asian	3	1.2			1	1.7	1	1.2		
Hispanic	2	0.9			0		2	2.4		
Black	1	0.3			0		1	1.2		
Indigenous	28	8.3			12	10.2	6	7.2		
**Marital Status**										
Single	75	27.7			16	27.1	28	32.9	1	10.0
Married or living with partner	191	70.5			42	71.2	57	67.1	9	90.0
Divorced or widowed	5	1.8			1	1.7	0	0	0	0
Household Income level**										
Under $25,000	24	8.7			3	5.1	6	7.1	0	
$26,000–$60,000	86	31.8			21	35.5	23	27	5	50.0
$61,000–$100,000	130	47.3			31	52.5	32	37.7	5	50.0
Over $100,000	31	11.4			4	6.8	24	28.2	0	
**Do you have any children under the age of 18?**										
No	144	53.1			29	49.2	57	67.1	6	60.0
Yes	127	46.9			30	50.8	28	32.9	4	40.0
**Are you pregnant or breastfeeding?**										
No	205	75.6			45	76.3	75	88.2	4	40.0
Yes	65	24			14	23.7	10	10.8	6	60.0
**How often do you smoke cigarettes, including e-cigarettes and vape products?**										
Never	156	57.6			35	58.3	70	83.3	2	20.0
Infrequently	72	26.6			18	30.0	9	10.7	2	20.0
Weekly	22	8.1			4	6.7	1	1.2	3	30.0
Daily	19	7.0			3	5.0	4	4.8	3	30.0
How often do you use cannabis (including edibles)? ***										
Never	175	64.6			42	70.0	48	57.1	6	60.0
Infrequently	75	27.7			16	26.6	24	28.6	3	30.0
Weekly	14	5.2			2	3.3	7	8.3	1	10.0
Daily	5	1.8			0		5	6.0	0	
**How often do you drink alcohol?**										
Never	67	24.7			16	26.7	26	31.0	0	
Once week or less	133	49.1			35	58.3	38	45.2	7	70.0
2–4/week	51	18.8			7	11.7	15	17.9	2	20.0
5–7/week	13	4.8			2	3.3	1	1.2	1	10.0
> 1/day	6	2.2			0		4	4.8	0	

Veg, Vegetarian; GF, Gluten free. *Participants were able to select multiple ethnicities under a Canadian identity. **Income in Canadian dollars. ***Cannabis use is legal in Canada.

Overall, the majority of respondents (68.6% of 271) rated their health as at least good or better while 6% rated their health as ‘bad’. Additional information about participants’ general medical history can be found in Supplementary Table 1.

### Participant beliefs

The information on participant beliefs and behaviours is presented using mixed analysis for the entire sample as well as by diet pattern (note that the subgroups vegetarian and GF or vegan and GF were collapsed into either vegetarian or vegan for the diet pattern breakdowns). Three main themes related to participant beliefs along with a number of related sub-themes were found. Health and ethical/moral concerns were the overarching themes related to the ‘why’ of the self-selected exclusionary dietary choice while the third theme of social judgement was related to the perception (both from self and others) of their dietary choices. Sub-themes for health included: nutrition, body image/ideal, disease risk, weight, allergies, and pregnancy. Sub-themes for ethical/moral concerns included animal welfare/rights, environmental, and individual choice. Sub-themes for social judgement included: criticism, guilt, hesitancy, and isolation. A small proportion of responses did not fit into any of the main themes or sub-themes and were categorized as alternate responses.

#### The meaning of following vegan, vegetarian, and gluten-free diets

Health was the main reason given (40.4% out of *n* = 222 who answered this question) for following a vegan, vegetarian, and/or GF diet. One respondent said that ‘*It means I eat more well rounded and really have to be cautious of what I am putting into my body’.* Meanwhile, another individual said that their diet *‘Means better cholesterol levels and lower blood pressure, both major risk factors for heart disease’.* Animal welfare and the environment were the primary sub-themes (20.2%) under ethical and moral concerns. One respondent wrote *‘I’m an ethical vegan. I do not purchase or consume animal products because they are the result of suffering and violence’,* while another said that by following their diet, they were *‘taking one step towards a kinder world where humans and animals peacefully co-exist with each other’.* Thirty-three percent of respondents reported that the meaning of their dietary choice was to either partially or totally eliminate specific foods or food groups. Almost 6% saw the diet as part of their identity or *‘faith’.*

#### Dairy and egg consumption

**Figure 2.**
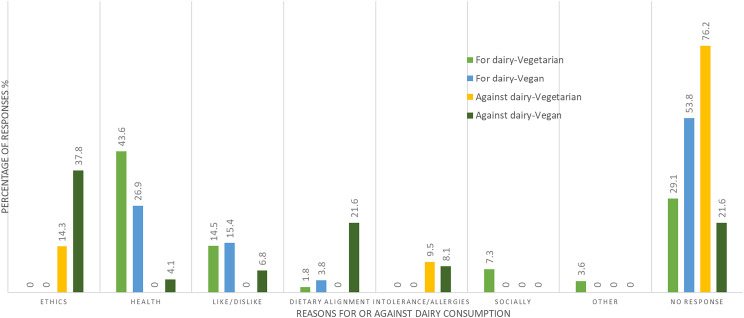
Reasons for or against dairy consumption given by Canadians capable of bearing children. *N* = 271.

A majority (54.6%) of the respondents indicated they consumed dairy. (See Table 2). Reasons for or against dairy consumption are examined in detail in Figure 2. Of the 153 participants who gave reasons, more vegetarians (*n* = 24) indicated they consumed dairy for health reasons as they commented repeatedly on the essential nutrients and protein content found in dairy products. Conversely, more vegans focused on the negative aspects of health, like dairy has *‘too many calories’* and that it is ‘*[…] associated with a variety of diseases including cancer, heart disease, obesity, and some auto immune disorders’* as reasons to avoid dairy. Some participants (9.2%) indicated an intolerance or allergy to dairy that prevented them from consuming dairy, while more vegans than vegetarians avoided it based on their dietary alignment convictions. Eighty-four percent of respondents who gave animal or environmental ethical concerns as their reason for avoiding dairy were vegan. One participant indicated exclusion *‘To avoid supporting the dairy industry because it is inherently cruel plus it’s bad for the environment’.* A small proportion of vegetarians (7.3%) indicated they will consume dairy in social situations or for the sake of convenience.

**Table 2.** Dietary patterns and beliefs of Canadians capable of bearing children (*n* = 271)

	*Total*		*Veg*		*Vegan*		*GF*
*Dietary patterns and beliefs*	*n*	*%*	*n*	*%*	*n*	*%*	*n*
Do you feel any hesitation to tell others about your current diet?							
No	199	73.4	48	81.4	60	70.6	9
Yes	67	24.7	10	16.9	25	29.4	1
Not Provided	5	1.8					
How does your current diet compare to the Canada’s Food Guide Recommendations https://food-guide.canada.ca/en/							
Not at all alike	16	5.9	0		6	7.1	0
Slightly alike	69	25.5	13	22	22	25.9	2
Moderately alike	94	34.7	29	49.2	19	22.4	3
Very alike	56	20.7	12	20.3	19	22.4	4
Extremely alike	14	5.2	1	1.7	7	8.2	1
Not Provided	22	8.1					
Do you eat eggs? If yes, how often?							
No	137	50.5	19	32.2	73	86.9	5
Yes	129	47.6	39	66.1	11	13.0	5
*Every day*	22	16.9					
*3-6 times per week*	16	12.3					
*1-2 times per week*	13	10.0					
*< 1 time per week*	5	3.8					
Not Provided	5	1.8					
Do you consume dairy products?							
No	119	43.9	11	18.6	70	82.4	3
Yes	148	54.6	47	79.7	15	17.6	7
Not Provided	4	1.5					
How do you feel following your current diet?							
Somewhat/extremely dissatisfied	29	11.3	3	5.1	2	2.4	1
Neither satisfied nor dissatisfied	27	10.0	2	3.4	1	1.2	2
Somewhat satisfied	122	45.0	34	57.6	29	34.1	6
Extremely satisfied	85	31.4	18	30.5	51	60	1
Not Provided	8	3.0					
Do you plan to follow this diet long term?							
No	35	12.9	5	8.5	3	3.5	1
Yes	225	83.0	52	88.1	80	94.1	8
Not Provided	11	4.1					
If pregnant or lactating, did your diet change upon becoming pregnant/breastfeeding?							
No	163	60.1	39	66.1	54	63.5	7
Yes	63	23.2	11	18.6	5	5.9	3
Not Provided	45	16.6					

Veg, Vegetarian; GF, Gluten-free.

Egg consumption was almost evenly split between consumers and non-consumers (Table 2). Two-thirds of the vegetarians indicated they consumed eggs; however, 13 % of those who identified as vegan indicated they also consumed eggs because *‘*[it’s a] *protein supplement’* or because *‘if it calls for it’.*

#### Reasons for beginning/following vegan, vegetarian, and gluten-free diets

When asked why they began following an exclusionary diet, 19.2% of the 166 respondents who answered this question referred to a combination of three factors: animal welfare, health, and the environment. Twelve percent noted a transition over time for beginning the diet versus the current reason(s) sustaining their dietary choices. For example, one individual said: *‘I began the diet because I was struggling with disordered eating and initially transitioned to veganism as a means to restrict my food intake. I was vegan for 4 years and then moved to vegetarianism because I realized my decision to become vegan was driven by disordered eating tendencies and I wished to incorporate more protein-containing foods in my diet. Now though, I find meat, chicken, and fish unappealing’.* Health remained a prominent theme (30.7%) for starting a vegan/vegetarian or GF diet (e.g. *‘Once I became more cognizant of my health[,] I wanted to do everything I can to reduce disease/illness, much of which is heightened by consumption of animal products’*. For those respondents focused on the sub-theme of weight/ideal body (13.9%), they believed that the exclusionary diet would help them achieve the ‘*perfect*’ body. Other sub-themes were animal welfare (10.8%), ethics (5.4%), and preference (4.8%). Two individuals (1.2%) commented that they follow their diet(s) because of pregnancy with one person saying, *‘I was pregnant three months ago[,] so I had to follow the diet’.* Additionally, one respondent specified beginning their diet for environmental sustainability: *‘I am a land-use scientist. Crunching the numbers, the impact of a plant-based diet is around a quarter (depending on what resource you’re looking at) of a standard western diet. From a sustainability perspective, it is massively impactful’.*

#### Hesitation to disclose dietary patterns

When asked if they felt any hesitation to tell others about their current diet, 24.7% (*n* = 67) answered ‘yes’ (Table 2). Of these individuals, 34.3% indicated feeling judgement, stigma, and criticism from others. One respondent explained: *‘People make fun of vegans or have judgements about me being pregnant or feeding our children vegan diets. I’d rather not hear it’.* Respondents also commented about situations in which people get defensive about their own choices (6.0%) after hearing that the CCBC follows an exclusionary diet pattern. One person said, ‘*people don’t like to be reminded of the wrong things they do, saying I don’t eat animals most people become defensive’.* Others were hesitant to share their dietary habits with others because of the need for accommodations or inconvenience (7.5%). One respondent wrote *‘I don’t like to inconvenience large groups or new people I’m meeting’.* There was also a feeling of guilt that prevented one participant from telling others about their diet: *‘[I] feel very guilty and embarrassed that I don’t have the courage of my convictions to fully eliminate eggs [.]’.* One respondent also commented on how their geographical location made them hesitant to disclose their diet because a ‘*vegan gluten free diet is a pretty extreme diet in rural Alberta*’.

#### Reasons for following diet long-term

Most respondents, both vegetarian and vegan, want to continue with their choice of diet for the long term (Table 2). When asked why, 42% of those who gave a reason (*n* = 143) answered health, but animal/environmental was also a common reason (19.3%), especially among those who identified as vegan. One person said, *‘it’s not a diet, but a moral stance’*. Interestingly, one person who identified as vegan/GF said *‘Yes, but it can be exhausting to always have to read labels, find ingredient substitutions, etc. I am not always strict with eating gluten free and occasionally “cheat” because it is simply exhausting to have to think so deeply* [….]*’.* Of those who said they do not plan to follow the diet long-term, 14 participants gave reasons. One person who identified as vegetarian said, *‘I want to be fully vegan soon’* while another who identified as vegan said they were not going to continue with their diet choice because of *‘fear of malnutrition’.*

#### Beliefs surrounding pregnancy and dietary choices

One question (Table 2) specifically asked respondents about their dietary pattern and pregnancy or breastfeeding, although the topic of pregnancy did come up several times in various questions as comments. When asked about what else they would like to share about their diet, 5.1% out of 121 who answered this question, wrote about a desire and/or ability to maintain their diet throughout pregnancy, breastfeeding, and/or intention to raise their children according to their diet. One commented, *‘If/when I get pregnant in the future, I would do everything I could to maintain my plant-based diet’.* A second wrote, *‘I have two daughters that I maintained my vegan gf diet throughout both pregnancies and both breastfeeding journeys (was pregnant/breastfeeding for a continuous 6 years without any negative impact)’.*

Almost one quarter of respondents revealed that their diet changed upon becoming pregnant or breastfeeding (Table 2). The primary reason for this was related to different nutritional requirements (33.3%), such as ensuring a balanced diet or increasing protein to support the baby. Two people mentioned their diet changed during pregnancy because of cravings, where they *‘increased dairy due to cravings, [because they are] easy nutrients*’.

#### Beliefs around dietary pattern, foods, and health

One hundred and forty-eight participants responded to the question about beliefs around animal products and health (Table 3). Health concerns, either with the food or elements within the products, were the predominant beliefs surrounding animal-based foods. Conversely, 6.8% believed that animal products were not harmful to health when consumed in moderation. Sixty-nine responded to the question of beliefs about gluten and their health (Table 3). Beliefs about gluten and health varied between no benefit to health and benefits to health although some participants reported bloating as a common gastrointestinal effect from consumption.

**Table 3.** The beliefs of Canadians capable of bearing children around dietary pattern, foods and health (*n* = 271)

Animal products in vegetarian/vegan diet	%	Example citation
Removing them improves health	26.0	*‘Animal products contribute to a multitude of diseases and conditions that can be reversed by eliminating them from your diet’.*
Have negative physiological impact	14.9	*‘milk makes you secrete mucus […] and meat upsets my stomach […]’.*
Concerns around component of animal products	9.1	‘*I believe that the cholesterol, saturated fat, and heme iron* [found in animal products] *are terrible for human beings’.*
		*‘they* [animal products] *can increase cholesterol and often unhealthy fats and pollutants such as heavy metals or hormones’.*
Detriment of specific animal products	6.8	‘*Animal products like meat and fish are unhealthy because you consume the animal’s blood, pus, hormones, and the antibiotics they were pumped full off. Dairy and eggs aren’t the healthiest but it’s not as bad as meat’.*
Unnecessary to health	13.5	*‘I don’t think milk is meant for kids over age 5, it’s natural to self wean by then’.*
		*‘They serve no real purpose because all nutrient[s] can be subbed through plant-based means…’.*
**Gluten in GF diet**	**%**	**Example citation**
Not sure about connection between gluten and health	15.9	*‘Handmade gluten is hygienic’.*
Good/bad/no effect on health	24.6	*‘Gluten-free diets don’t seem to have any healthy benefits’.*
Cause adverse gastrointestinal symptoms	17.4	‘*Makes me bloat excessively, sometimes painful’.*
Impact on physical appearance	8.7	*‘make me slimmer’.*
		*‘make my skin better’.*
Differentiated between types of gluten-containing foods	7.2	‘*believe that wheat products available in the current market are not good quality*’.

GF, Gluten-free.

### Participant behaviours

Eighty-three percent of those who identified as vegetarian reported adhering to their dietary pattern 75% to 100% of the time (Table 4). Almost all (97.2%) of those who identified as vegan reported adhering most to all of the time. Those who declared they followed a GF diet were more variable. More participants answered the question about dietary adherence than those who answered the original question about which diet they followed. Most respondents (85.3%) indicated they never cheat on their diet or if they do, it is by accident (Table 4). Those who intentionally cheat often did so because they were eating at a restaurant or not at home (18.5%), while more vegetarians than vegans occasionally cheat because of being pregnant. Further information on behaviours surrounding self-selected dietary patterns is found in Supplementary Table 2.

**Table 4.** Diet adherence and behaviours of Canadians capable of bearing children (*n* = 271)

*Diet Adherence/Behaviours*	*Total*	*%*	*Veg*		*Vegan*		*GF*
	*n*	*%*	*n*	*%*	*n*	*%*	*n*
How strictly do you follow the diet?							
Vegetarian							
Never intentionally follow (0%)	12	4.4	2	3.3			
Rarely follow (25%)	17	6.3	1	1.7			
Sometimes follow (50%)	61	22.5	5	8.3			
Frequently follow (75%)	68	25.1	22	36.7			
Strict 100% of the time	88	32.4	28	46.7			
Vegan							
Never intentionally follow (0%)	8	3.0			0	0	
Rarely follow (25%)	26	9.6			0	0	
Sometimes follow (50%)	48	17.7			2	2.4	
Frequently follow (75%)	94	34.7			23	27.4	
Strict 100% of the time	87	32.1			59	70.2	
Gluten Free							
Never intentionally follow (0%)	54	20.0					1
Rarely follow (25%)	44	16.2					0
Sometimes follow (50%)	69	25.5					3
Frequently follow (75%)	52	19.2					4
Strict 100% of the time	25	9.2					1
If you ever ‘cheat’ on your diet, is it most often:							
Intentional	27	10.0	5	8.3	10	11.9	1
Accidental	142	52.4	31	51.7	37	44.0	7
I never cheat	89	32.8	17	28.3	33	39.3	2
Not Provided	13	4.8					
What are your reasons for intentionally ‘cheating’?							
Eating at a restaurant/out of the home	50	18.5	16	26.7	12	14.3	3
Someone else is cooking for me	45	16.6	1	1.7	4	4.8	1
To gain additional nutrients from the diet	34	12.5	3	5.0	2	2.4	1
Pregnant/lactating	31	11.4	9	15	3	3.6	3
Other	10	3.7	2	3.3	8	9.5	0
I do not intentionally cheat	88	32.5	24	40.0	52	61.9	2
Not Provided	13	4.8					
Do you ever feel tempted to ‘cheat’?							
No	204	75.3	46	76.7	59	70.2	7
Yes	60	22.1	11	18.3	25	29.8	3
Not Provided	7	2.6					
Do others in your life follow the same diet as you? Why or why not?							
No	114	42.1	21	35.0	37	44.0	5
Yes	147	54.2	36	60.0	46	54.8	5
Not Provided	10	3.7					

Veg, Vegetarian. GF, Gluten-free.

#### Adherence to exclusionary diets by social circle

**Figure 3.**
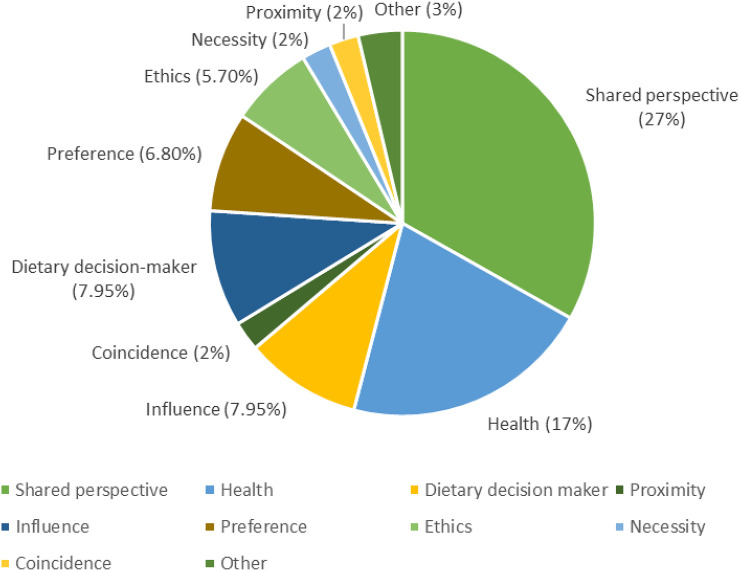
Reasons offered why others in the lives of Canadians capable of bearing children follow the same dietary patterns. *N* = 271.

The majority of respondents indicated that others in their lives also followed the same diet (Table 4). As can be seen Figure 3, several themes emerged from participant responses (*n* = 94) with the most frequent reasons being shared perspective on dietary matters (27%) and health (17%). One respondent wrote ‘*because they know it’s best for their health*’. Other factors that emerged included: being the dietary-decision-maker for the household (8.0%), influence over others (8.0%), preference for diet (6.8%), ethics (5.7%), coincidence (3%), necessity (2%), and proximity or convenience (2%). Fifteen participants did not give a reason.

**Figure 4.**
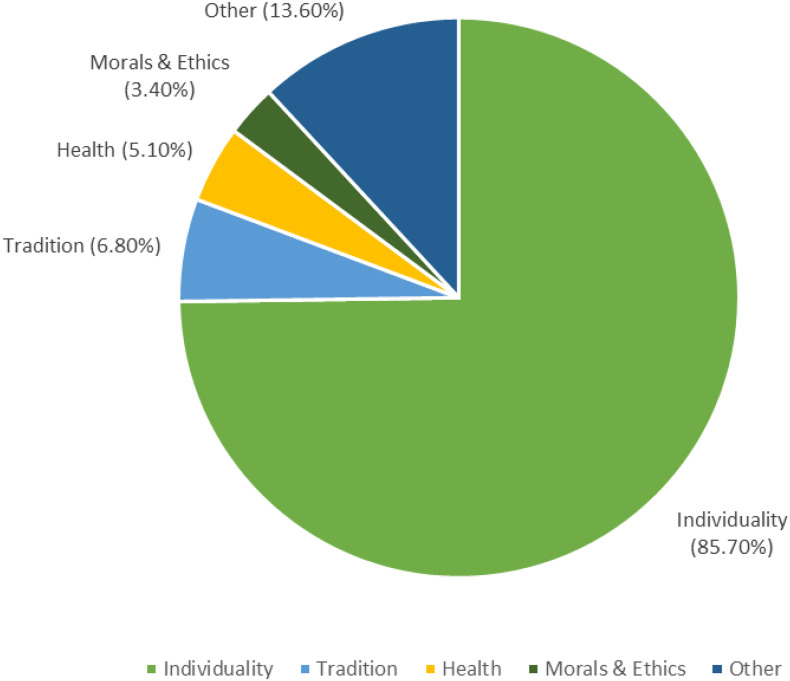
Reasons offered why others in the lives Canadians capable of bearing children do not follow the same dietary patterns. *N* = 271.

Conversely, 43.7% said that others in their life do not adhere to the same diet, but the reasons for this were far fewer (Figure 4) with most respondents (73 respondents gave reasons) identifying the concept of individuality or individual choice as the reason for not following the same dietary pattern. One participant wrote *‘They can make their own choices. I do what’s best for me and my body’,* while another said, *‘Don’t use your own ideas to constrain each other’.* Adhering to cultural traditional around eating meat was another sub-theme that emerged (6.8%), which prevented others in their life from following a vegetarian or vegan diet. A small group (3.4%) highlighted the lack of morals in others as a reason for not following the dietary pattern (e.g. *‘because they are morally bankrupt’*).

#### Impact of diet(s) on life

When asked if their life was impacted in any way by their self-selected dietary choice(s), 178 participants, 41% of whom identified as vegan, answered the open-ended question. A small number (16.5%) indicated their dietary choices had not been impacted, but the majority indicated their lives were impacted in some way. The area in their lives most impacted (15.4%) was around relationships. Some noted gains in relationships, such as finding community with others who follow the same diet (e.g. *[…] I have […] fostered a community with similarly minded people*); others discussed relationship losses (e.g. *‘I don’t talk to my family anymore […]’.*). Some participants (6.5%) reported experiencing ostracization from family, friends, and colleagues, saying ‘[I’ve*] been socially excluded by my family and pressured severely to eat meat’*. Others have faced challenges from others (e.g. *‘[…] I have faced backlash from people that I lost respect for […]’).* Interestingly, the proportion of vegans and vegetarians reporting being ostracized was the same (6.8%). After relationships, a general impact from their dietary choice(s) was reported by (14.3%) of the group. This included impacts on their activity levels and overall health, with the discussion centring on increased activity and a general sense of feeling healthier. Others reported changes to activities (6.3%) like hobbies, eating out less often versus cooking more and cooking more healthfully, or needing to plan (shopping, eating, activities) more (7.4%). Inconvenience (primarily to others) was also reported by a small group as having a negative impact on the lives of the CCBC.

## Discussion

The aim of this study was to explore the beliefs and behaviours of CCBC who follow vegetarian, vegan, and/or GF diets using mixed methods. While there is a growing body of research examining the nutritional adequacy of these diets, their impact on health and, more recently, the impact on maternal/foetal outcomes, there remains a dearth of research looking at the explicit beliefs held by CCBC that determine dietary choices and behaviours during the reproductive years outside of the state of pregnancy.

The results of this study show three overarching themes framing the beliefs of CCBC in relation to their dietary choice. Health was the primary factor, with individuals seeking to make themselves healthier, or avoiding the compounds in animal-based or gluten-containing foods associated with negative health effects like chronic disease or allergies. Second to health were the moral and ethical concerns surrounding the foods not consumed in the chosen dietary pattern. Animal welfare, environmental concerns and individual choice were captured under this overarching theme. The last overarching theme found in the present research was that of social judgement, primarily from others about the dietary choices, but also from the participants themselves. This included the negative perceptions of criticism, guilt, hesitancy, and isolation/ostracization.

Participants were found to have strong beliefs in the ability of their self-selected exclusionary diet pattern to impart health, either by the avoidance of animal-containing or gluten-containing foods and/or the inclusion of healthier foods and activities. The emphasis on health by most of the sample aligns with past literature where health has frequently been identified as a strong driver of individual motivation to follow exclusionary diets.^(7,8,32)^ With respect to vegetarian and vegan diets, CCBC pointed to the ability of these diets to reduce disease and chronic illness risk. Other research has reported similar findings; Lea and Worsley, reported that 89% of vegetarians believed that vegetarianism can prevent general disease.^(32)^

The beliefs about the healthful properties of their self-selected diet are generally supported by the literature. Studies such as the longitudinal cohort EPIC-Oxford Study which looked at vegetarian/vegan diets over time, showed risk is reduced for diabetes, cardiovascular disease (CVD), and some cancers, but conversely, increased for bone fractures and stroke.^(33)^ Recent systematic reviews/meta-analysis and umbrella reviews have also shown these risk reductions. Improvements in total cholesterol, low density lipoproteins, but not triglycerides,^(34,35)^ glycated haemoglobin,^(35,36)^ BMI,^(35,36)^ and insulin sensitivity^(36)^ have all been found with vegetarian and vegan diets. Risks for CVD^(37,38)^ and ischaemic heart disease^(37,38)^ are reduced. However, inconsistent evidence exists for stroke. Some studies report reductions in ischaemic stroke^(38)^ but not total stroke.^(37)^ Even a short exposure to a healthy vegan diet, such as 8 weeks, has been shown to improve cardiometabolic outcomes.^(39)^ The most recent position paper from the Academy of Nutrition and Dietetics summarizes the evidence as low to moderate for vegetarian diets to reduce risk for the above conditions but shows potential harm for bone health.^(6)^ There is less evidence for the effects of a vegan diet on health.

The protective effect that vegetarian/vegan diets have against disease may be related to the nutrient quality of these diets compared to omnivorous diets. Many CCBC noted their concern over the compounds found in animal products as a reason to maintain a vegetarian or vegan diet. Almost 6% of CCBC believed that dairy contributes to the development of various diseases/conditions; conversely, about 44% of those who identified as vegetarians indicated that they believed dairy to be healthy and nutritious. This disparity is common in the literature with one study from Belgium finding that 55.3% of vegetarians disagreed with the idea that milk was healthy.^(40)^

Our findings suggest that CCBC who do not consume dairy products are primarily motivated by animal and environmental ethics. Vegetarians are less likely to be motivated by animal and environmental concerns compared to vegans for reducing or eliminating dairy consumption.^(41)^ Interestedly, 15 people who identified as vegan indicated they consumed dairy with one person saying *‘there is milk powder in the chips I like. I feel I do enough to minimize animal cruelty that I can rationalize the small amount*’. It is also possible that the use of the word ‘dairy’ in the question may have caused confusion since dairy-free milks/beverages are also found in the refrigerated dairy section of the grocery store in Canada. Tailoring education to address diverse motives may enhance the effectiveness of nutrition information.

Respondents also referred to their desire to continue their diets during current or future pregnancies. Given the importance of one-carbon nutrients during this life stage, there may be concern for nutrient deficiencies. While the 2025 Academy Position Paper^(6)^ does not discuss pregnancy/lactation, unlike the 2016 Position Paper,^(4)^ there is evidence that well-balanced vegetarian and vegan diets can generally meet the nutrient and energy needs of this crucial period, especially if supplements are used regularly.^(14,15)^ Generally, there is more concern with vegan diets increasing the risk of lower birth weights in newborns, although the inconsistency of the evidence at this time limits the ability to draw firm conclusions.^(16,17,27)^ This suggests that healthcare professionals may need to provide appropriate resources about dietary choices and supplementation to CCBC who follow these self-selected exclusionary diet patterns and are planning to become pregnant.

Beyond pregnancy, CCBC also discussed choosing to raise their children to follow the same dietary practices. In some CCBC households, being the dietary-decision-maker was one reason noted as to why their children also eat vegetarian, vegan, and/or GF diets. This finding is supported by recent studies.^(42,43)^ Parents decide together on the type of diet given to the child, driven primarily by ethical reasons.^(43)^ Importantly, there was an element of shared decision-making wherein some mothers would allow their children to eat meat/meat products if they wanted to,^(42,43)^ the idea of individual choice shared by some CCBC in the present study. As one participant stated, *‘husband is not vegan, but hubby[and] parents[are] happy to eat vegan, and 2/3 kids [are] vegetarian’.*

Ethical concerns over environmental sustainability and animal welfare were part of the ethical/moral overarching theme that was repeated throughout the questions and is in keeping with past literature exploring motives to follow vegetarian and vegan diets. For example, Ruby and colleagues found that European-American vegetarians were significantly more likely to be concerned with the daily impact of their food choices on both environmental and animal welfare as compared to omnivores.^(44)^ Conversely, Corrin and Papadopoulos report that animal welfare and environmental sustainability were ranked lowest among non-health-related benefits of vegetarian and vegan diets.^(13)^ Additionally, there may be differences in the attitudes towards animal welfare between vegetarians and vegans.^(45)^ The current study supports these attitudes with health being the primary driver of vegetarians who consume dairy versus the ethical concerns of those who identify as vegan. As one individual said, *‘I think all animals are the same and need to be protected. Why love a dog but eat a pig’?*

Interestedly, dietary adherence does seem to be shaped by whether the primary driver of the dietary choices is based in either health or ethical concerns. We found that approximately one third of the sample indicated following a vegetarian and/or vegan diet 100% of the time, and almost half of the sample followed their chosen dietary pattern between 50-75% of the time. Evidence suggests that vegans are significantly more likely to be motivated by ethical concerns, as compared to health,^(25)^ and that vegetarians who are motivated by ethical concerns tend to practice greater dietary restriction compared to health motivated vegetarians.^(46)^ It should be noted that the numbers reported in the dietary adherence questions do not necessarily align with how many indicated their dietary pattern. However, 41% did not indicate their dietary pattern at the beginning of the survey and may have been captured within this question.

CCBC reported that their dietary habits required greater accommodation or inconvenienced others. This is consistent with the literature where there is a general belief that vegetarian diets are inconvenient^(13)^ or they are a burden if they follow a GF diet.^(47)^ These beliefs may negatively impact dietary adherence, as adherence is supported when surrounded by others who accommodate dietary choices.^(48,49)^ The current study supports this notion as 35% of CCBC admitted to cheating on their diet because they were not eating at home or because someone else was cooking for them. Furthermore, it is also possible that because they feel like an inconvenience, CCBC may practice more flexibility in their diet (i.e. ‘cheating’) as part of a negotiation strategy to confront eating-related social norm conflicts.^(50)^

The idea of inconvenience/accommodation and negotiation further influenced eating behaviours around shopping and eating out. Some CCBC compensated by cooking more (10.5%) and eating out less (9.6%), and 11.2% mentioned they need to plan more. These types of behaviour changes have been previously reported by adults following GF^(23)^ and/or vegan^(21)^ diets. In the current study, more vegans than vegetarians reported eating out less (12.3% vs 6.8%) and planning more (17.8% vs 6.8%). There was an expressed wish by a number of CCBC that the surrounding food environment was more supportive of their dietary choices. This is echoed in the literature where 65% of Australian study participants reported limited choice when eating out as a barrier to vegetarian diets.^(51)^ More CCBC vegans than vegetarians (88% vs 51.7%) ask if their meal at a restaurant follows their dietary alignment and further ask about ingredients. As one participant said, *‘[I ask] every time, I don’t trust them. I have been served something or told something was vegan, and when asked for them to confirm that, it was indeed not vegan’.* Relatedly, 42% of the sample admitted to telling servers that they have food allergies, even when they do not, just to avoid certain foods. This practice may be problematic, especially in the context of a GF diet, as the ‘faddishness’ of the diet may undermine the medical needs of those diagnosed with CD.^(52)^ However, geographic differences may be responsible for the expressed idea of a more supportive food environment. One respondent noted an abundance of vegan establishments in the urban area where they resided, which is likely different from the options available to the respondent who indicated living in rural Alberta where a vegan diet would be viewed as extreme outlier.

The beliefs held by CCBC also included the idea of social judgement. This was a minor but concerning theme that crossed several of the questions we asked. A number of CCBC revealed feeling hesitant to disclose their dietary habits to others. One reason for this was fear of the judgement that may come with discussing dietary choices. Relatedly, some CCBC also described experiencing ostracization from family, friends, and colleagues because of their diet(s) with both vegetarians and vegans reporting this. A review of the literature assessing attitudes and perceptions towards plant-based diets found that female vegetarians received hostility and disapproval about their diet, especially from meat-eating male family members.^(13)^ These negative experiences may be explained by the fact that vegetarians and vegans do not conform to a society where eating meat is considered ‘normal’, thus making them a target of bias.^(53)^ There is also the negative stereotype that vegans are believed to be hostile and confrontational.^(13)^ Several of the CCBC reported that others became defensive when the CCBC disclosed their dietary choice, increasing reluctance to disclose dietary choices in the future. A GF diet does not exclude CCBC from these experiences, with research suggesting that those who adhere to a GF diet feel ‘stigmatized’, ‘left out’, and ‘embarrassed’.^(52)^ This discriminatory behaviour from omnivores/others towards those who follow these self-selected exclusionary diet patterns thus entails a ‘social cost’ and may increase the perception of being outsiders.^(54)^ Healthcare providers need to be aware of their own biases and prejudices surrounding these dietary patterns to improve communication and reduce distrust of omnivore healthcare professionals.

To our knowledge, the current study is one of the first to capture the voice of CCBC and explores some of the whys behind their decision-making surrounding their dietary choices.

The study has several limitations. First, the participants were primarily white, educated, with moderate- to high-incomes which may have limited the generalizability of our results. However, it is important to note that these demographic characteristics largely fit with what has been reported previously,^(7,23,25,26,52)^ suggesting that our sample is typical of those following a vegetarian, vegan, or GF diet in Canada. Second, recruitment was conducted on social media, which may have introduced a self-selection bias, and limited our sample to those who frequent these platforms. However, individuals who follow these dietary patterns often seek community with others who share their interests and beliefs, and social media plays a vital role in facilitating these communities and also increases the likelihood of reaching CCBC across the country.^(46)^ Selection bias might have also been introduced by the offered incentive, which may have increased non-responsiveness by those not interested in the survey itself.^(30,55)^ The survey design allowed participants to ‘skip’ questions (other than the required consent question) which may explain why 108 submissions had little to no information. While evidence shows that incentives can increase data quality, there is always the possibility of either increased socially desirable answers or careless responses, so careful scrutiny of the data is recommended.^(30,55)^ Lastly, a high proportion of the population did not indicate if they followed a vegetarian, vegan or GF diet. Participants were asked to fill in their diet pattern which led to a broader range of answers (see Table 1) but also meant a high proportion (41%) did not give an answer. This may be a weakness in how the survey was created and may lower the reliability of the data collected. However, the survey did have 102 questions, many of which were open text allowing us to capture information about respondent’s beliefs about their dietary choices which may not have otherwise been expressed.

## Conclusion

This study assessed the beliefs and behaviours of CCBC who choose to follow a vegetarian, vegan, and/or GF diet(s) which can help healthcare practioners to better understand how to frame nutrition information while still respecting the personal choices of their patients. Health was an important factor that influenced belief surrounding the dietary patterns of CCBC, though not all beliefs were based in science. Ethical concerns around animal welfare and the environment were found to especially influence those who followed a vegan dietary pattern. A concerning theme around the negative elements of social judgement, primarily targeted at the participants but also from the participants themselves towards others in their social sphere suggests that there may be a ‘social cost’ to following their self-selected dietary pattern. Future research should continue to explore how beliefs shape behaviour with self-selected dietary patterns but also explore this concept of social costs and how geographical differences could further exacerbate social judgement, especially of those individuals isolated in rural areas.

## Supporting information

Morello et al. supplementary material 1 Morello et al. supplementary material

Morello et al. supplementary material 2 Morello et al. supplementary material

## Supplementary material

To view supplementary material for this article, please visit https://doi.org/10.1017/jns.2025.10044.
