# Supplementary material for: Beliefs and behaviours associated with vegetarian, vegan, and gluten-free diets among Canadians capable of bearing children
Source: J Nutr Sci. 2025 Oct 14;14:e73. doi: 10.1017/jns.2025.10044 (PMC12554814; doi:10.1017/jns.2025.10044)
Supplement: Morello et al. supplementary material 1 — Morello et al. supplementary material [file S204867902510044Xsup001.docx]

**Supplementary Table 1.** General medical history of respondents (n=271)

| *Medical History* | *n* | *%* |
| --- | --- | --- |
| **Do you use contraceptives?** |  |  |
| No | 159 | 59.0 |
| Yes | 110 | 40.6 |
| Not Provided | 2 | <1.0 |
| **Do you use any medications regularly?** |  |  |
| No | 202 | 74.5 |
| Yes | 68 | 25.1 |
| Not Provided | 1 | <1.0 |
| **Does your medical history include any of the following illnesses/conditions?** |  |  |
| Unresolved gastrointestinal symptoms | 84 | 31.0 |
| Diagnosed gastrointestinal disorder | 44 | 16.2 |
| *Celiac disease* | 2 | 4.5 |
| *Inflammatory bowel disease* | 3 | 6.8 |
| *Other* | 5 | 11.4 |
| *Response not provided* | 34 | 77. |
| Diagnosed irritable bowel syndrome | 33 | 12.2 |
| Other malabsorption/metabolic/excretion disorders. | 12 | 4.4 |
| *Iron deficiency anemia* | 2 | 16.7 |
| *Constipation* | 2 | 16.7 |
| *Other* | 2 | 16.7 |
| *Response not provided* | 6 | 50.0 |
| **If an illness/condition was selected in the previous question, who diagnosed you with this illness/condition?** |  |  |
| Specialized physician (E.g. gastroenterologist) | 84 | 45.2 |
| Family Doctor | 43 | 23.1 |
| Alternative practitioner (E.g. naturopath, holistic  nutritionist) | 11 | 5.9 |
| Family/friend | 20 | 10.8 |
| Self-diagnosed | 20 | 10.8 |
| Other | 8 | 4.3 |
| **Do you have any other diagnosed or suspected illnesses?** |  |  |
| No | 216 | 79.7 |
| Yes | 49 | 18.1 |
| *Mental health* | 15 | 30.6 |
| *Eczema* | 4 | 8.2 |
| *Asthma* | 3 | 6.1 |
| *Fibromyalgia* | 2 | 4.1 |
| *Arthritis* | 3 | 6.1 |
| *Other* | 16 | 32.7 |
| *Response not provided* | 6 | 12.1 |
| Not Provided | 6 | 2.2 |
| **How would you describe your overall health?** |  |  |
| Extremely bad | 3 | 1.1 |
| Moderately bad | 13 | 4.8 |
| Slightly bad | 31 | 11.4 |
| Neither good nor bad | 37 | 13.7 |
| Slightly good | 42 | 15.5 |
| Moderately good | 97 | 35.8 |
| Extremely good | 47 | 17.3 |
| Not Provided | 1 | <1.0% |
| **Do you have any health complaints?** |  |  |
| No | 207 | 76.4 |
| Yes | 58 | 21.4 |
| *Pain* | 13 | 22.4 |
| *Mental health* | 4 | 6.9 |
| *Malnutrition* | 5 | 8.6 |
| *Fatigue* | 3 | 5.2 |
| *Gastrointestinal-related complaints* | 7 | 12.1 |
| *Other* | 5 | 8.6 |
| *Response not provided* |  |  |
| Not Provided | 6 | 2.2 |
| **Do you have any food allergies or intolerances?** |  |  |
| No | 205 | 75.6 |
| Yes | 65 | 24.0 |
| *Lactose intolerance* | 18 | 27.7 |
| *Shellfish/seafood* | 4 | 6.2 |
| *Gluten/celiac* | 7 | 10.7 |
| *Peanuts/tree nuts* | 6 | 9.2 |
| *Other* | 18 | 29.2 |
| *Response not provided* | 12 | 18.5 |
| Not Provided | 1 | <1.0 |

No anthropometric data was collected.
