# Supplementary material for: Beliefs and behaviours associated with vegetarian, vegan, and gluten-free diets among Canadians capable of bearing children
Source: J Nutr Sci. 2025 Oct 14;14:e73. doi: 10.1017/jns.2025.10044 (PMC12554814; doi:10.1017/jns.2025.10044)
Supplement: Morello et al. supplementary material 2 — Morello et al. supplementary material [file S204867902510044Xsup002.docx]

**Supplementary Table 2**. Behaviors around self-selected dietary patterns in Canadians capable of bearing children.

| *Behaviours* | *Total* | *%* | *Veg* |  | *Vegan* |  | *GF* |
| --- | --- | --- | --- | --- | --- | --- | --- |
|  | *n* | *%* | *n* | *%* | *n* | *%* | *n* |
| Do you purchase gluten free/vegan alternatives to regular foods? |  |  |  |  |  |  |  |
| No | 137 | 50.6 | 30 | 50.0 | 15 | 17.9 | 9 |
| Yes | 130 | 48.0 | 28 | 46.7 | 68 | 81 | 1 |
| Not Provided | 4 | 1.5 |  |  |  |  |  |
| Are there any non-food products you avoid? (ex. gluten-containing or non-vegan lip balm) |  |  |  |  |  |  |  |
| No | 136 | 50.2 | 35 | 58.3 | 26 | 31.0 | 6 |
| Yes | 130 | 48.0 | 23 | 38.3 | 57 | 67.9 | 4 |
| Not Provided | 5 | 1.8 |  |  |  |  |  |
| Do you check ingredient lists when you consume packaged food? |  |  |  |  |  |  |  |
| No | 72 | 26.6 | 7 | 11.7 | 7 | 8.3 | 3 |
| Yes | 196 | 72.3 | 51 | 85.0 | 77 | 91.7 | 7 |
| Not Provided | 3 | 1.1 |  |  |  |  |  |
| When eating at a restaurant, do you ask if your meal is gluten free/vegan/vegetarian? |  |  |  |  |  |  |  |
| No | 105 | 38.7 | 27 | 45.0 | 9 | 10.7 | 7 |
| Yes | 161 | 59.4 | 31 | 51.7 | 74 | 88.1 |  |
| Not Provided | 5 | 1.8 |  |  |  |  |  |
| When eating at a restaurant, do you ask for the ingredients in each dish? |  |  |  |  |  |  |  |
| No | 167 | 61.6 | 38 | 63.3 | 48 | 57.1 | 6 |
| Yes | 101 | 37.3 | 20 | 33.3 | 36 | 42.9 | 4 |
| Not Provided | 3 | 1.1 |  |  |  |  |  |
| Do you tell wait staff that you have allergies to avoid certain foods (even though you do not have true allergies)? |  |  |  |  |  |  |  |
| No | 99 | 36.5 | 21 | 35.0 | 42 | 50.0 | 3 |
| Yes | 113 | 41.7 | 25 | 41.7 | 22 | 26.2 | 6 |
| Sometimes | 56 | 20.7 | 12 | 20.0 | 20 | 23.8 | 1 |
| Not Provided | 3 | 1.1 |  |  |  |  |  |
| If a food contains minimal amounts of gluten-containing or animal-derived ingredients, do you eat it anyways? |  |  |  |  |  |  |  |
| No | 145 | 53.5 | 36 | 60.0 | 52 | 61.9 | 2 |
| Yes | 49 | 18.1 | 7 | 11.7 | 8 | 9.5 | 5 |
| Sometimes | 74 | 27.3 | 15 | 25.0 | 24 | 28.6 | 3 |
| Not Provided | 3 | 1.1 |  |  |  |  |  |

Veg, Vegetarian. GF, Gluten-free.
